# Supplementary material for: Metabolic Pathway Modeling in Muscle of Male Marathon Mice (DUhTP) and Controls (DUC)—A Possible Role of Lactate Dehydrogenase in Metabolic Flexibility
Source: Cells. 2023 Jul 25;12(15):1925. doi: 10.3390/cells12151925 (PMC10417281; doi:10.3390/cells12151925)
Supplement: Supplementary file 1 [file cells-12-01925-s001.zip › cells-2476518 revision_Brenmoehl et al-suppl.Fig_proofread.pdf]

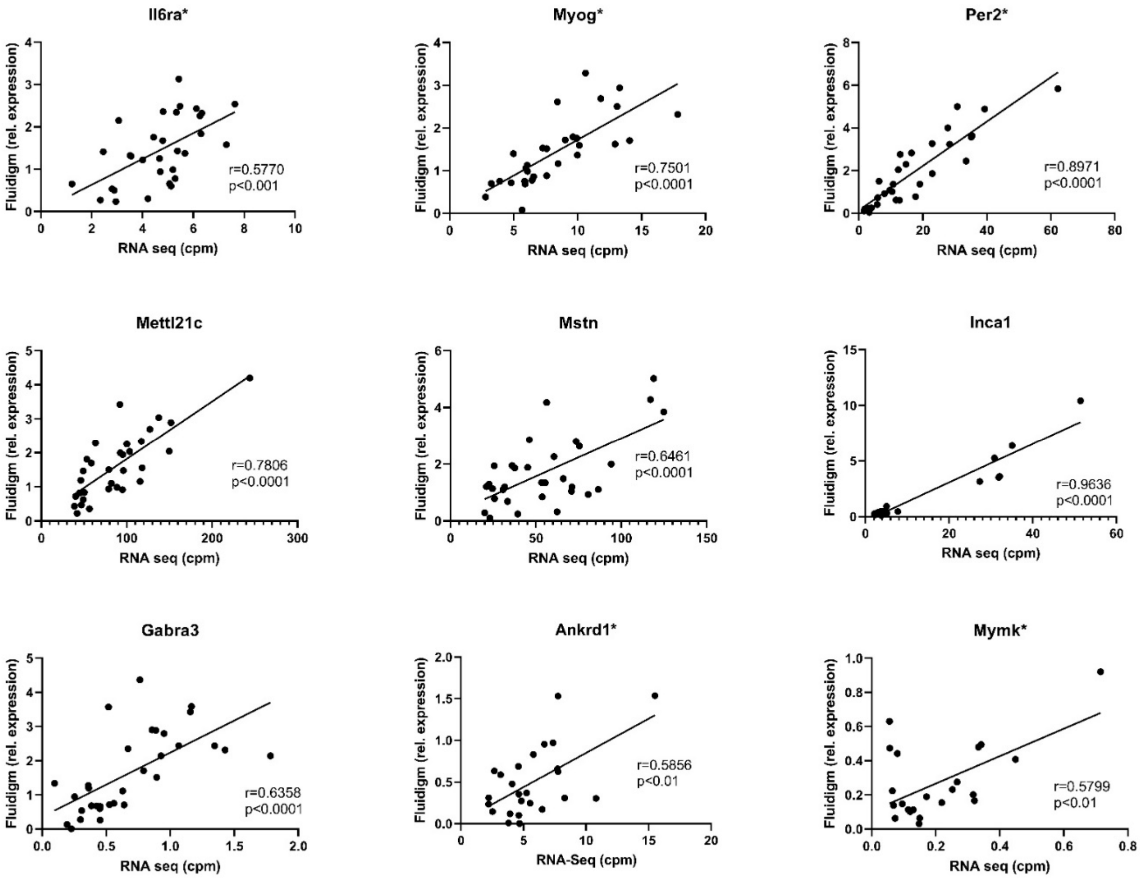

Figure S1: Validation of RNA-seq data by the Fluidigm technique for nine differentially expressed genes (DEGs). For each gene, the total reads (count per million, cpm) obtained by RNA-seq were plotted on the x-axis and RT-qPCR data ($2^{-\Delta\Delta Ct}$) on the y-axis. Stars at the gene name indicate that outliers were removed during processing, as described in Materials and Methods. Corresponding correlation coefficients (r) and p-values are shown. Abbreviations: Ankrd1 – Ankyrin repeat domain-containing protein 1, Gabra3 – Gamma-aminobutyric acid A receptor subunit alpha 3, Il6ra – Interleukin 6 receptor alpha, Inca1 – Inhibitor of CDK, Cyclin A1 interacting protein 1, Mettl21c – methyltransferase like 21C, Mstn – myostatin, Mymk – myomaker, MyoG – myogenin, Per2 – period circadian clock 2.

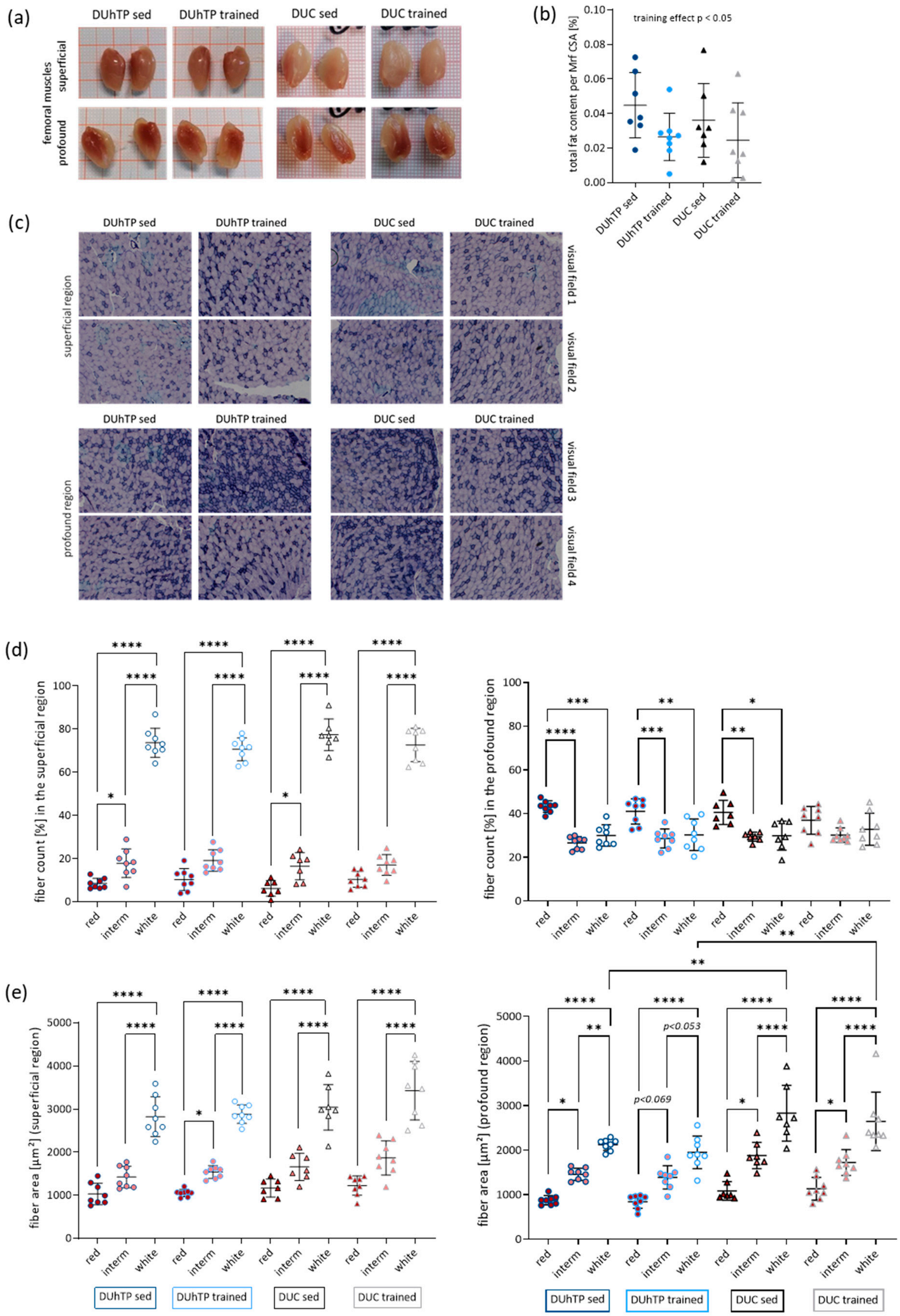

Figure S2: Immunohistochemical evaluation of *Musculus rectus femoris* (Mrf) of trained and sedentary (sed) DUhTP and DUC mice at the age of 70 days (n = 7-8 animals). **a)** Representative dissected Mrfs showing superficial region (upper panel) and profound (lower panel) region of sedentary and trained DUhTP (left) and DUC mice (right). **b)** Percentage of total fat per cross-sectional area (CSA) in the four groups. The training-mediated effect was statistically evaluated by using the unpaired t-test with Welch correction. **c)** Representative histological images of

10 μm Mrf cryosections stained with NADH-tetrazolium reductase to determine red (dark blue), intermediate (mid blue), and white (light blue) fibers in sedentary and trained DUhTP (left) and DUC (right) mice. Shown are two images, each from the superficial and profound regions from a mouse of each group. **d)** Percentage muscle fiber count and **e)** muscle fiber area per μm^2 analyzed muscle region (see Method part) of red (dark red), intermediate (light red), and white fibers (white) in trained and sedentary DUhTP (light/dark blue) and DUC (gray/black) mice were measured and calculated by using by image analysis. Data are shown as scatter plots with means and standard derivations. Statistical analysis was performed using the linear model followed by TukeyHSD post hoc test to test the dependence between phenotype and the interaction of line x treatment x muscle fiber type. Significant differences as indicated: * $p < 0.05$, ** $p < 0.01$, *** $p < 0.001$, **** $p < 0.0001$. Borderline significances as indicated.

(a) DUhTP sedentary vs. DUC sedentary DUhTP trained vs. DUC trained

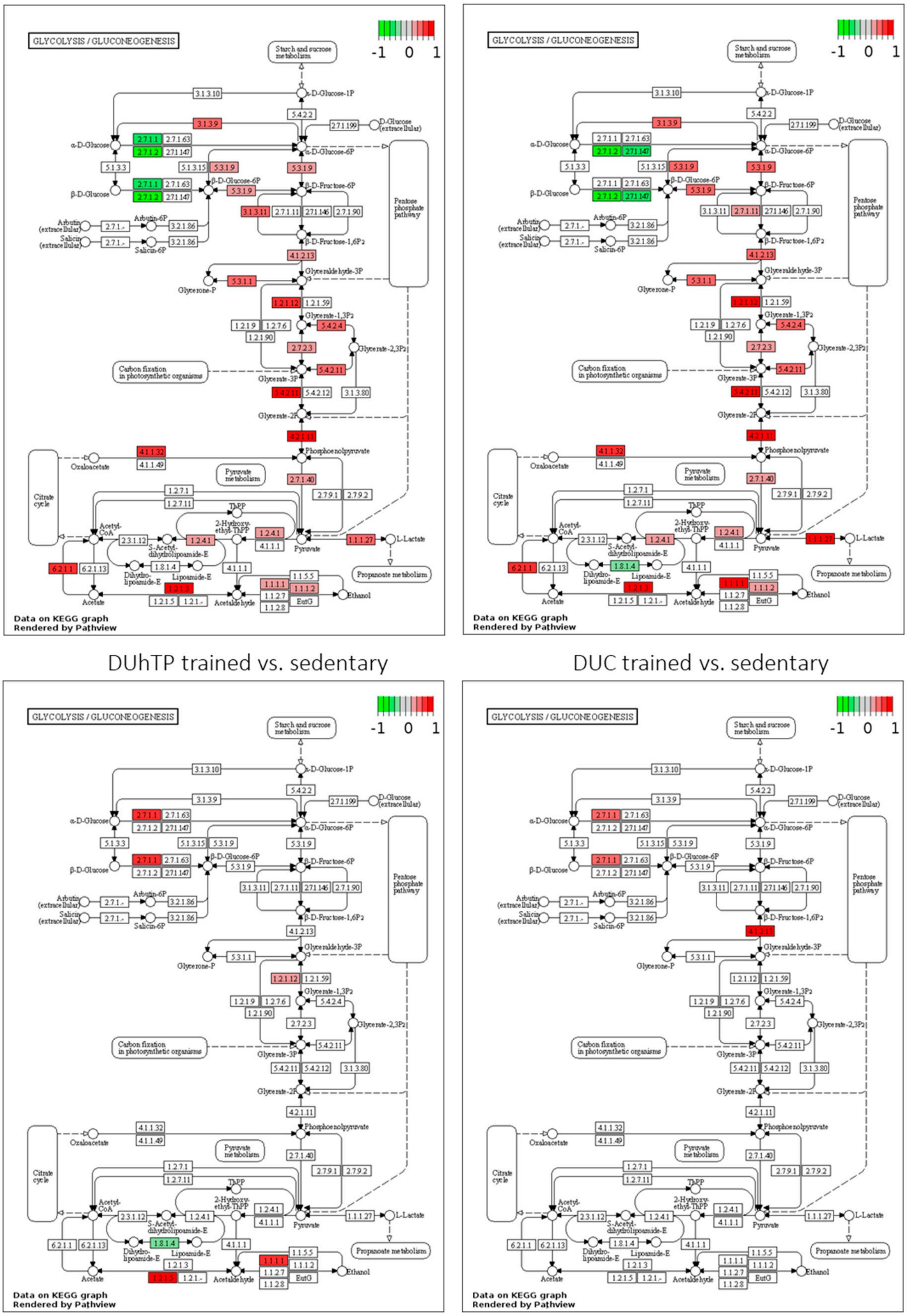

(b)

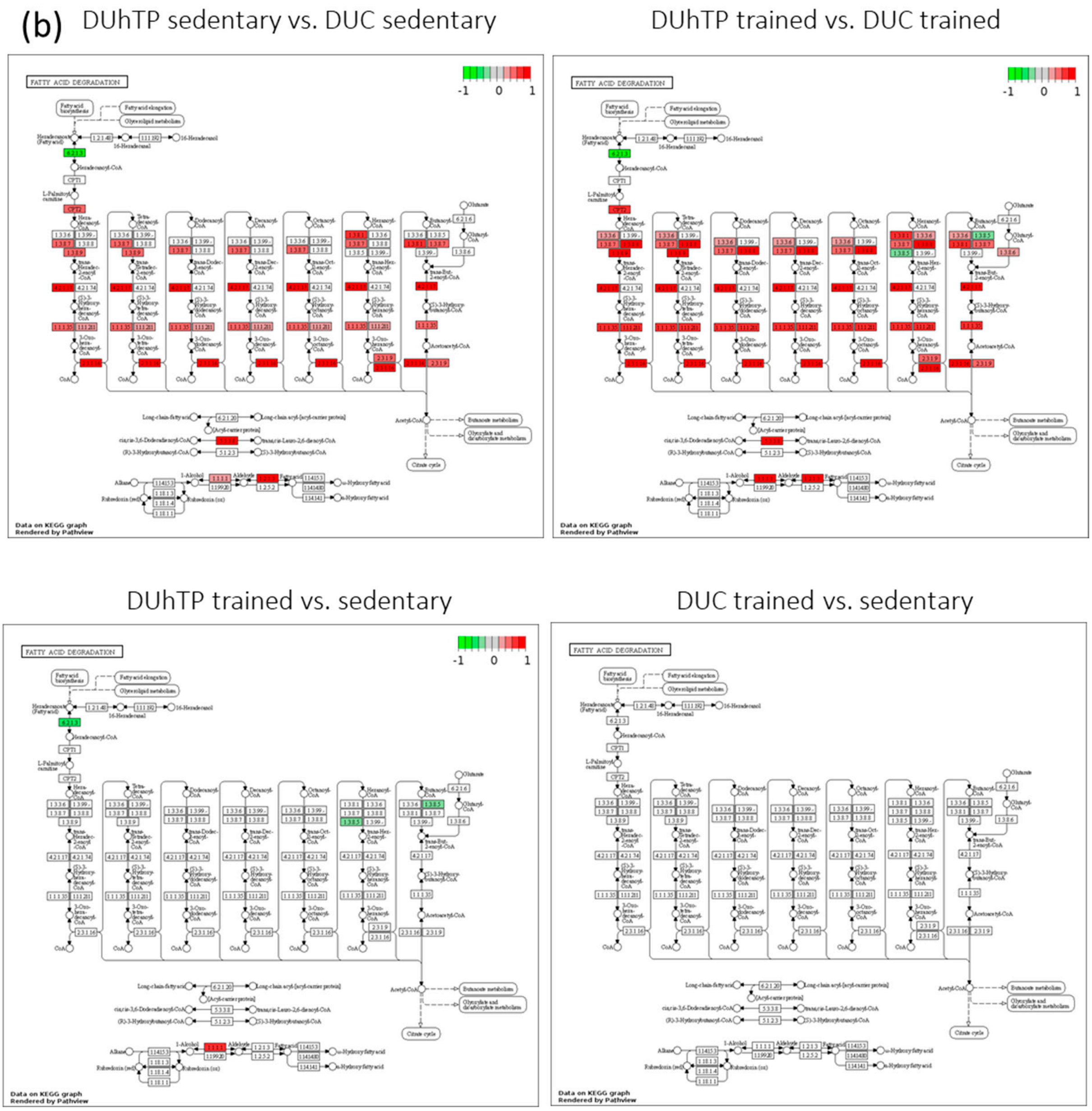

DUhTP sedentary vs. DUC sedentary
DUhTP trained vs. DUC trained
DUhTP trained vs. sedentary
DUC trained vs. sedentary
FATTY ACID DEGRADATION
-1 0 1
Data on KEGG graph
Rendered by Pathview

(c)

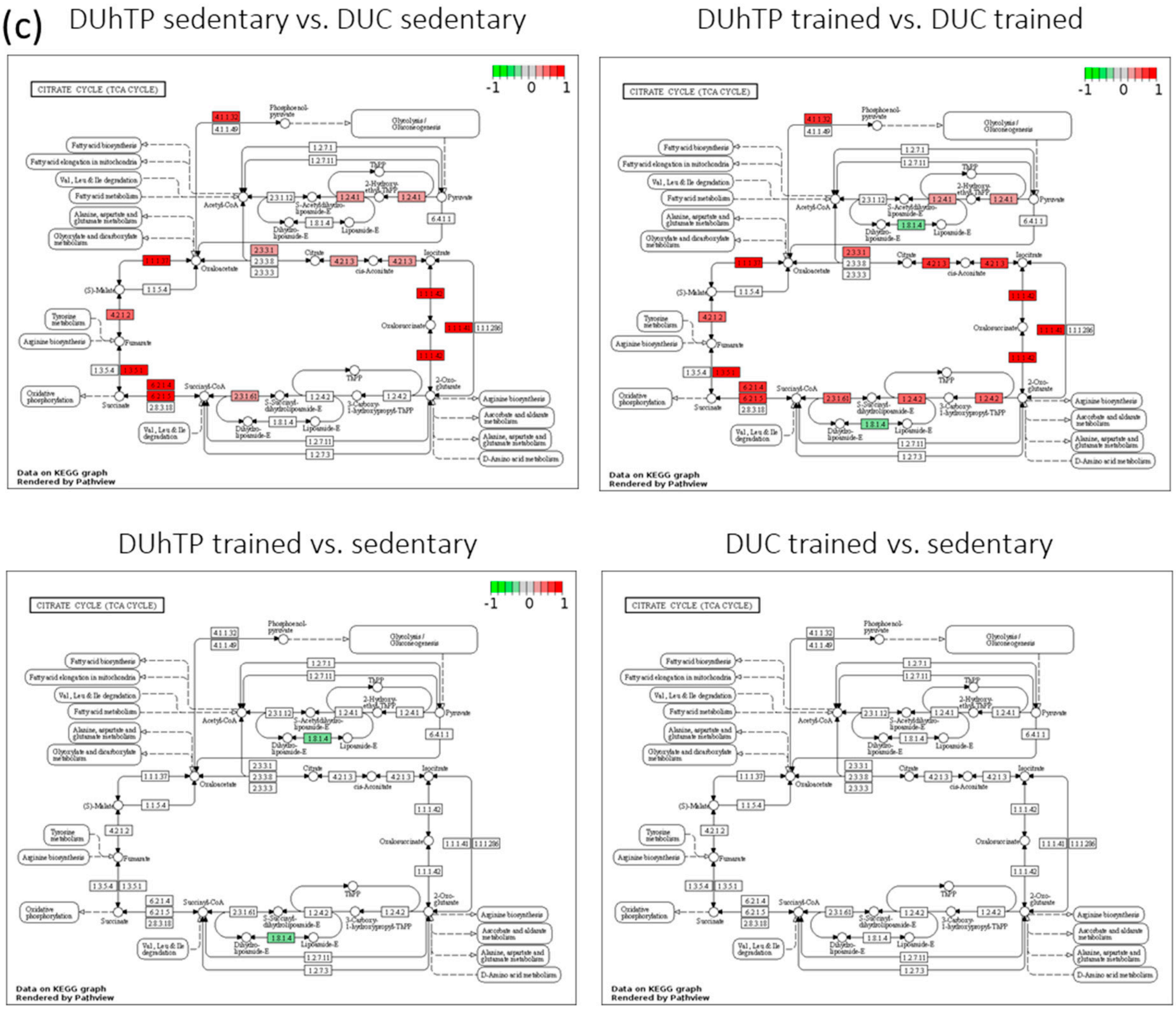
DUhTP sedentary vs. DUC sedentary
DUhTP trained vs. DUC trained
DUhTP trained vs. sedentary
DUC trained vs. sedentary
CITRATE CYCLE (TCA CYCLE)
-1 0 1
Data on KEGG graph
Rendered by Pathview

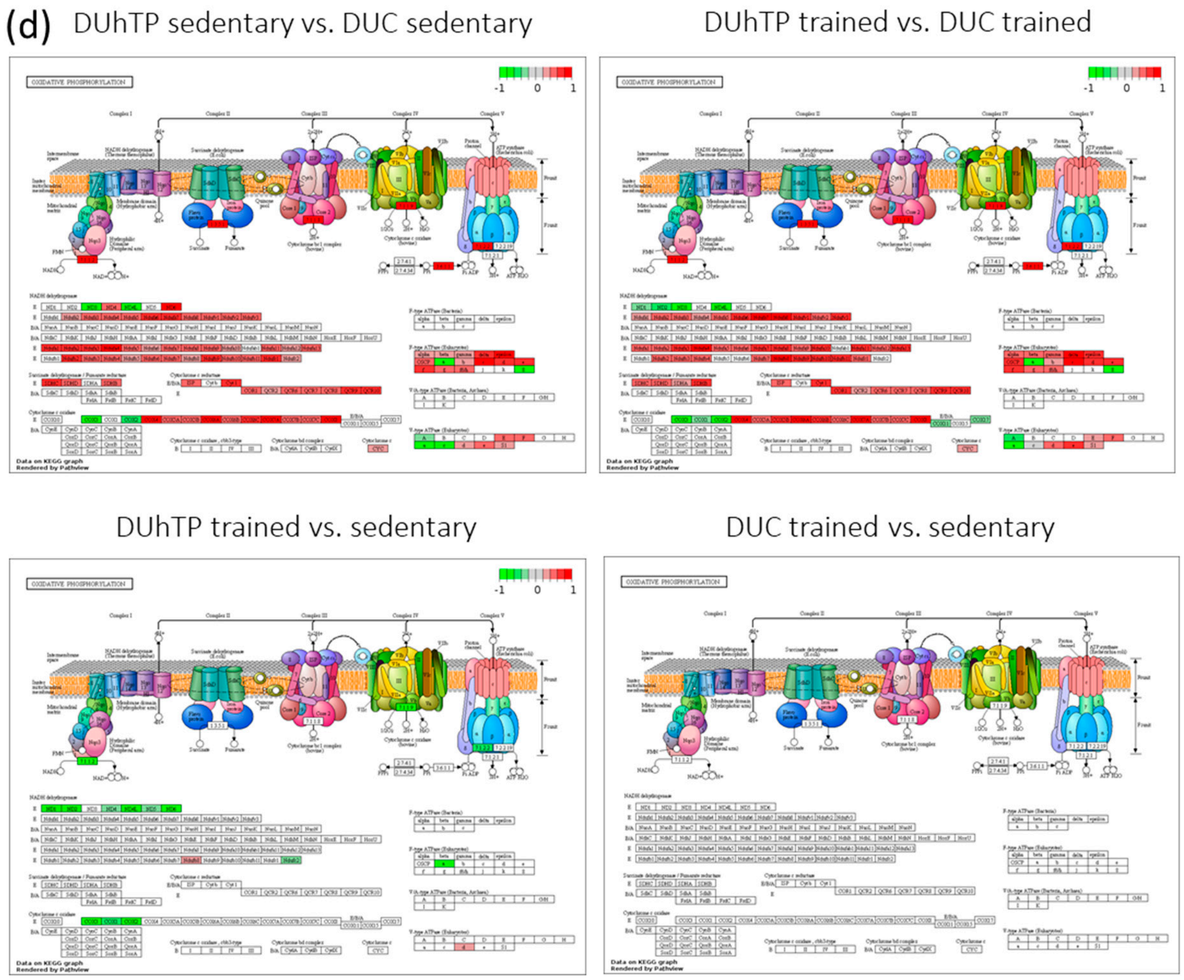

Figure S3: Graphical visualization of gene regulation in a) glycolysis, b) fatty acid degradation, c) TCA cycle, and d) oxidative phosphorylation KEGG pathways for the four comparison groups. The KEGG Pathway analyses were obtained by using the DEGs (FDR < 0.05) of all comparison groups (DUhTP sedentary vs. DUC sedentary, DUhTP trained vs. DUC trained, DUhTP trained vs. sedentary, DUC trained vs. sedentary) via https://pathview.uncc.edu (access date: 12/01/2022). Abbreviation: DEGs = differential expressed genes, vs. = versus

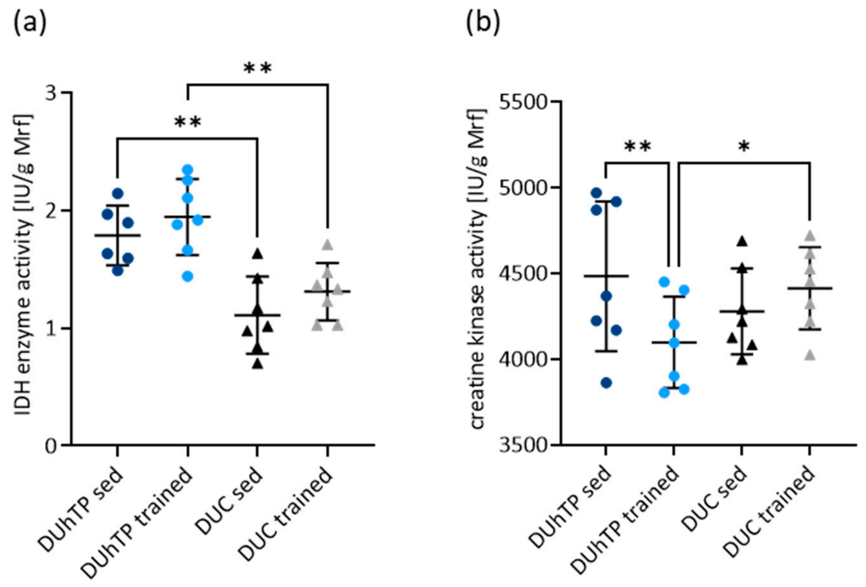

Figure S4: Analysis of a) isocitrate dehydrogenase (IDH) enzyme activity and b) creatine kinase enzyme activity per gram *Musculus rectus femoris* of trained and sedentary DUhTP (light/dark blue) and DUC mice (gray/black). Results are shown as scatter plots with mean and standard derivations. Statistical analysis was performed using two-way ANOVA. Significant differences are marked with: * $p < 0.05$, ** $p < 0.01$, **** $p < 0.0001$.
