# Supplementary material for: Metabolic Pathway Modeling in Muscle of Male Marathon Mice (DUhTP) and Controls (DUC)—A Possible Role of Lactate Dehydrogenase in Metabolic Flexibility
Source: Cells. 2023 Jul 25;12(15):1925. doi: 10.3390/cells12151925 (PMC10417281; doi:10.3390/cells12151925)
Supplement: Supplementary file 1 [file cells-12-01925-s001.zip › cells-2476518 revision_Brenmoehl et al-suppl.Tab_proofed.pdf]

Table S1: Body weight, weight gain, and food consumption of all mice during the 3-week experiment, starting from day 49 and ending at day 70. All data are means with standard deviations and were statistically analyzed using two-way ANOVA with GraphPad. Different letters denote significant age-related differences ($p < 0.05$). Hashtags mark significant differences compared to sedentary or trained control mice DUC ($p < 0.05$), and paragraph symbols mark significant differences compared to sedentary littermates of the respective line ($p < 0.05$).

	Experiment time	DUhTP sed n = 19	DUhTP trained n = 19	DUC sed n = 17	DUC trained n = 23
Body weight [g]	day 49	30.67 ± 2.31^{a}#	30.07 ± 2.86^{a}#	35.47 ± 4.37^{a}	37.07 ± 2.66^{a}
	day 56	31.90 ± 2.61^{b}#	30.23 ± 3.04^{a}#	36.99 ± 4.14^{b}	37.78 ± 2.66^{b}
	day 63	32.83 ± 2.77^{c}#	30.92 ± 3.03^{b}#	38.48 ± 4.39^{c}	38.69 ± 2.67^{c}
	day 70	34.21 ± 2.60^{d}#	31.04 ± 2.73^{b}#§	39.35 ± 4.44^{d}	39.18 ± 2.94^{c}
Weight gain [g]	week 1	1.24 ± 0.90	0.29 ± 0.95§	1.52 ± 0.80^{a}	0.70 ± 0.69§
	week 2	0.93 ± 0.84	0.58 ± 0.54	1.50 ± 0.55^{a}	0.91 ± 0.70§
	week 3	1.37 ± 0.97	0.09 ± 1.14§	0.86 ± 0.58^{b}	0.49 ± 1.10
weekly feed intake [g]	week 1	35.23 ± 2.90^{a}#	36.48 ± 3.21^{a}#	51.15 ± 10.19	45.03 ± 7.90
	week 2	38.01 ±3.55^{b}#	39.83 ± 3.64^{b}#	47.57 ± 4.53	44.80 ± 5.21
	week 3	36.65 ± 4.04^{a}#	38.96 ± 7.62^{a,b}#	49.48 ± 6.01	45.87 ± 6.85

Table S2: Primer sequences for quantitative real-time PCR for selected genes.

Gene	forward primer 5′→3′	reverse primer 5′→3′
Actb	TGACAGGATGCAGAAGGAGA	CGCTCAGGAGGAGCAATG
Ankrd1	GCTGGTAACAGGCAAAAAGAAC	CCTCTCGCAGTTTCTCGCT
Gabra3	CTCTCTGCTTCGGGGAAGTG	CTTGGCTAGTGGTTCCAGGG
Il6ra	CCTGAGACTCAAGCAGAAATGG	AGAAGGAAGGTCGGCTTCAGT
Inca1	ATGCCTCAGCCGTATGGAGAT	GCCCTCAGAATTGGTGGAATGTA
Mettl21c	ACTCTCGGGGACTCCACAG	GCTCTTGTGTGTAGCTGGCATA
Mstn	AGTGGATCTAAATGAGGGCAGT	GTTTCCAGGCGCAGCTTAC
Mymk	TTCCTCCCGACAGTGAGCAT	GCACAGCACAGACAAACCAG
MyoG	AGTGAATGCAACTCCCACA	CTGGGAAGGCAACAGACATA
Per2	CAGAGGAGAAGACTCCGCAC	TTGCTGTCGCTGGATGATGT
Pgk1	CAGTCTAGAGCTCCTGGAAGGT	AGGAGCACAGGAACCAAAGG
Rplp2	GACGATGATCGGCTCAACAAG	ACCCTGAGCGATGACATCCT
Rpl26	GAACCGCAAACGGCATTTCA	TAGTGTCCGCGAACAACCTG

Abbreviations: Actb – actin β, Ankrd1 – Ankyrin repeat domain-containing protein 1, Gabra3 – Gamma-aminobutyric acid A receptor subunit alpha 3, Il6ra – Interleukin 6 receptor alpha, Inca1 – Inhibitor of CDK, Cyclin A1 interacting protein 1, Mettl21c – methyltransferase like 21C, Mstn – myostatin, Mymk – myomaker, MyoG – myogenin, Per2 – period circadian clock 2, Pgk1 – phosphoglycerate kinase 1, Rplp2 – ribosomal protein, large P2, Rpl26 – ribosomal protein L26. The housekeeping genes are underlined.

Table S3: List of all differentially expressed genes (DEGs) in the four different comparison groups, each group consisting of 8 animals, by RNAseq. Abbreviations: trained – 3-weeks treadmill training, sed – sedentary, FDR – false discovery rate

As an extra file

Table S4: Identification of differentially expressed genes (DEGs; FDR < 0.05) in the four different comparison groups, each group consisting of eight animals, by RNAseq, providing a) the number of total, upregulated, and downregulated transcripts. The percentage of upregulated or downregulated transcripts is noted in parentheses. b) List of enriched pathways for upregulated (upper part) or downregulated (lower part) genes in the comparison between trained DUhTP and DUC mice (DUhTP trained vs. DUC trained), ordered by the count of involved genes. The level of enrichment is reported as p-value and gene count. Abbreviations: trained – 3-weeks treadmill training, sed – sedentary

(a)

comparison group	DEGs	downregulated DEGs	upregulated DEGs
DUhTP sed vs. DUC sed	5916	3077 (52%)	2839 (48%)
DUhTP trained vs. DUC trained	7042	3751 (53%)	3291 (47%)
DUhTP trained vs. DUhTP sed	1606	1037 (65%)	569 (35%)
DUC trained vs. DUC sed	130	27 (21%)	103 (79%)

(b)

KEGG_PATHWAY	Count	Benjamini
**upregulated**		
Metabolic pathways	423	3.90E-27
Pathways of neurodegeneration - multiple diseases	172	8.00E-26
Amyotrophic lateral sclerosis	146	8.40E-26
Alzheimer disease	146	6.70E-24
Huntington disease	139	3.10E-32
Parkinson disease	134	3.20E-36
Prion disease	124	4.80E-29
Thermogenesis	116	5.10E-31
Chemical carcinogenesis - reactive oxygen species	109	3.70E-28
Diabetic cardiomyopathy	108	9.20E-30
Ribosome	106	3.20E-36
Oxidative phosphorylation	87	1.80E-33
Coronavirus disease - COVID-19	87	2.10E-11
Non-alcoholic fatty liver disease	78	1.30E-20
Salmonella infection	58	4.80E-02
Spliceosome	57	4.40E-11
Retrograde endocannabinoid signaling	54	1.20E-07
Carbon metabolism	51	1.10E-09
Spinocerebellar ataxia	45	1.20E-04
Biosynthesis of cofactors	42	6.20E-03
Fluid shear stress and atherosclerosis	38	3.30E-02
Autophagy - animal	37	3.30E-02
Peroxisome	35	4.00E-06
Cardiac muscle contraction	34	1.70E-05

Biosynthesis of amino acids	31	4.70E-05
Insulin resistance	30	4.00E-02
Proteasome	28	3.50E-09
Valine, leucine and isoleucine degradation	25	5.30E-05
Nucleotide metabolism	25	3.00E-02
Aminoacyl-tRNA biosynthesis	24	2.40E-03
Glycolysis / Gluconeogenesis	24	2.90E-03
Glutathione metabolism	24	8.00E-03
Mitophagy - animal	23	8.20E-03
Fatty acid metabolism	22	6.10E-03
Pyruvate metabolism	21	8.10E-05
Fatty acid degradation	21	1.40E-03
Citrate cycle (TCA cycle)	18	3.20E-05
Arginine and proline metabolism	18	3.50E-02
RNA polymerase	17	1.40E-04
Glyoxylate and dicarboxylate metabolism	15	2.80E-03
Glycine, serine and threonine metabolism	15	2.90E-02
Propanoate metabolism	14	6.60E-03
Fatty acid elongation	12	3.50E-02
2-Oxocarboxylic acid metabolism	10	2.30E-02
Sulfur relay system	7	4.40E-03
**downregulated**		
Herpes simplex virus 1 infection	142	2.50E-13
Pathways in cancer	128	1.40E-04
PI3K-Akt signaling pathway	83	4.20E-03
Human papillomavirus infection	83	4.80E-03
MAPK signaling pathway	74	9.40E-04
Endocytosis	69	1.20E-03
Salmonella infection	66	8.70E-04
Focal adhesion	62	1.40E-05
Regulation of actin cytoskeleton	60	6.80E-04
Lipid and atherosclerosis	59	6.80E-04
Proteoglycans in cancer	58	3.50E-04
Hepatitis B	56	1.50E-06
Rap1 signaling pathway	55	3.60E-03
Ras signaling pathway	54	3.00E-02
Autophagy - animal	51	1.50E-06
Transcriptional misregulation in cancer	51	4.30E-02

FoxO signaling pathway	49	1.20E-06
Protein processing in endoplasmic reticulum	49	9.00E-04
mTOR signaling pathway	46	8.70E-04
Signaling pathways regulating pluripotency of stem cells	45	1.40E-04
Axon guidance	45	1.60E-02
Cellular senescence	44	3.30E-02
Ubiquitin mediated proteolysis	42	2.30E-03
Insulin signaling pathway	41	1.40E-03
Hepatitis C	40	3.80E-02
Measles	39	9.00E-03
Growth hormone synthesis, secretion and action	38	5.30E-04
Yersinia infection	38	4.80E-03
Cell cycle	37	2.60E-03
Gastric cancer	37	3.90E-02
Prostate cancer	35	1.90E-04
Thyroid hormone signaling pathway	35	4.50E-03
Neurotrophin signaling pathway	35	4.80E-03
Sphingolipid signaling pathway	35	6.60E-03
Small cell lung cancer	34	1.40E-04
Nucleocytoplasmic transport	34	4.90E-03
Chronic myeloid leukemia	33	3.70E-06
Endocrine resistance	32	6.80E-04
TGF-beta signaling pathway	32	8.70E-04
Colorectal cancer	30	1.00E-03
Phosphatidylinositol signaling system	30	3.80E-03
mRNA surveillance pathway	30	9.00E-03
Toxoplasmosis	30	2.20E-02
AGE-RAGE signaling pathway in diabetic complications	29	1.30E-02
ErbB signaling pathway	28	2.30E-03
Longevity regulating pathway	28	5.20E-03
Lysine degradation	27	1.10E-04
EGFR tyrosine kinase inhibitor resistance	27	2.10E-03
PD-L1 expression and PD-1 checkpoint pathway in cancer	27	7.60E-03
ECM-receptor interaction	26	1.50E-02
Progesterone-mediated oocyte maturation	26	2.60E-02
Renal cell carcinoma	25	1.20E-03
Non-small cell lung cancer	25	2.60E-03
Pancreatic cancer	25	4.80E-03

Bacterial invasion of epithelial cells	25	4.80E-03
p53 signaling pathway	24	4.90E-03
Glioma	24	6.90E-03
Adherens junction	23	8.90E-03
Prolactin signaling pathway	23	1.50E-02
Viral life cycle - HIV-1	22	3.60E-03
Melanoma	22	2.20E-02
Long-term depression	21	5.70E-03
Longevity regulating pathway - multiple species	20	1.90E-02
Endometrial cancer	19	2.00E-02
Notch signaling pathway	19	2.80E-02
Hedgehog signaling pathway	18	4.20E-02

Table S5: List of significantly upregulated and downregulated levels of various acylcarnitines, di/triglycerides, phospholipids, and fatty acid derivates in serum samples of the four comparison groups (DUhTP sed vs. DUC sed, DUhTP trained vs. DUC trained, DUhTP trained vs. DUhTP sed, and DUC trained vs. DUC sed; each n = 9) shown as log2FC ($p < 0.05$). Metabolites were analyzed by high-resolution LC-MS/MS.

metabolite name	log2FC	p-Value	metabolite class
**DUhTP sed vs. DUC sed**			
Traumatic Acid	-4.37	0.000	Dicarboxylic Acid/ Fatty acid derivate (prostaglandin synthesis intermediate)
11-Aminoundecanoic acid	-3.74	0.000	Fatty acid/ Amine
Octatrienal	-2.15	0.000	Fatty aldehyde
7-Mercaptoheptanoylthreonine	-1.15	0.014	Fatty acid derivate
3-Hydroxytetradecanoic acid	1.47	0.022	Hydroxy fatty acid
10-Nitrooleate	1.48	0.019	Nitrated Fatty acid
9S,13R-12-Oxophytodienoic acid	1.59	0.033	Fatty acid derivate (lipoxygenase metabolite of linolenic acid)
5-Hydroxydecanoic acid	1.80	0.000	Hydroxy fatty acid
12-Oxododecanoic acid	1.88	0.008	Oxo fatty acid
3-oxopalmitic acid	1.89	0.048	Oxo fatty acid
12-Hydroxylauric acid	1.93	0.004	Hydroxy fatty acid
(2Z)-3,7-Dimethyl-2,6-octadien-1-yl 3-oxobutanoate	2.51	0.006	Fatty alcohol ester
2,3-Dinor-6-oxoprostaglandin F1alpha	2.84	0.000	Eicosanoide/ Prostaglandine/ arachidonic acid metabolite
3-Oxotetradecanoic acid	2.90	0.005	Oxo fatty acid
P-PE 41:6	-1.09	0.001	Phospholipid
SM 29:2 / sphingomyeline 29:2	-1.08	0.019	Phospholipid
PS 42:3 / phosphatidylserine 42:3	-1.03	0.000	Phospholipid
PC 37:2	-0.55	0.011	Phospholipid
PE 40:9	1.01	0.007	Phospholipid
1-[(9Z)-hexadecenoyl]-sn-glycero-3-phosphocholine (PC 18:1/20:3)	1.17	0.047	Phospholipid
TAG 48:2	1.06	0.006	Triglyceride
TAG 47:6	1.14	0.000	Triglyceride
(2S)-1-(Docosanoyloxy)-3-hydroxy-2-propanyl (7Z,10Z,13Z,16Z)-7,10,13,16-docosatetraenoate (DAG 22:0/22:5)	1.20	0.021	Diglyceride
TAG 62:16	4.74	0.003	Triglyceride
3-[(3-Hydroxydecanoyl)oxy]-4-(trimethylammonio)butanoate (3-hydroxydecanoyl carnitine)	1.16	0.027	Acylcarnitine
3-hydroxydodecanoylcarnitine	2.16	0.016	Acylcarnitine

9,12-Hexadecadienoylcarnitine	4.14	0.000	Acylcarnitine
**DUhTP trained vs. DUC trained**			
7-Mercaptoheptanoylthreonine	-2.26	0.000	Hydroxy fatty acid
11-Aminoundecanoic acid	-1.04	0.034	Fatty acid/ Amine
5-Hydroxydecanoic acid	1.02	0.003	Hydroxy fatty acid
12-Oxododecanoic acid	1.14	0.003	Oxo fatty acid
Brassylic acid	1.19	0.013	Dicarboxylic acid
12-Hydroxylauric acid	1.30	0.002	Hydroxy fatty acid
3-Hydroxytetradecanoic acid	1.52	0.001	Hydroxy fatty acid
3-oxopalmitic acid	1.70	0.050	Oxo fatty acid
DL-α-Aminocaprylic acid	1.79	0.000	Fatty acid/ Amine
(2Z)-3,7-Dimethyl-2,6-octadien-1-yl 3-oxobutanoate	2.00	0.004	Fatty alcohol esters
3-Oxotetradecanoic acid	2.11	0.001	Oxo fatty acid
P-PE 38:1	-1.61	0.026	Phospholipid
PC 37:2	-1.07	0.000	Phospholipid
PS 42:3 / phosphatidylserine 42:3	-0.85	0.000	Phospholipid
PS 40:0 /phosphatidylserine 40:0	0.79	0.049	Phospholipid
1-hexadecanoyl-2-(4Z,7Z,10Z,13Z,16Z,19Z-docosahexaenoyl)-sn-glycero-3-phosphocholine (PC (16:0/22:6))	1.22	0.025	Phospholipid
1-[(9Z)-hexadecenoyl]-sn-glycero-3-phosphocholine (LPC 16:1)	1.47	0.011	Phospholipid
TAG 48:2	0.88	0.027	Triglyceride
TAG 62:16	4.67	0.000	Triglyceride
2-Hexenoylcarnitine	-3.42	0.000	Acylcarnitine
Tiglylcarnitine	1.23	0.020	Acylcarnitine
Decanoylcarnitine	1.30	0.046	Acylcarnitine
3-[(3-Hydroxydecanoyl)oxy]-4-(trimethylammonio)butanoate (3-hydroxydecanoyl carnitine)	1.34	0.029	Acylcarnitine
Oleoylcarnitine	1.42	0.017	Acylcarnitine
3-hydroxydodecanoylcarnitine	1.51	0.014	Acylcarnitine
(2E)-hexadecenoylcarnitine	1.55	0.008	Acylcarnitine
3-Hydroxy-5, 8-tetradecadiencarnitine	2.11	0.014	Acylcarnitine
9,12-Hexadecadienoylcarnitine	2.21	0.044	Acylcarnitine
**DUhTP trained vs. DUhTP sed**			
2,3-Dinor-6-oxoprostaglandin F1alpha	-1.84	0.002	Eicosanoide/ Prostaglandine/ arachidonic acid metabolite
DL-α-Aminocaprylic acid	1.17	0.002	Fatty acid/ Amine

Icosanedioic acid	1.34	0.040	Fatty acid derivate dicarboxylic acid
Icosatetraenoic acid	1.49	0.025	Fatty acid
11-Aminoundecanoic acid	3.29	0.000	Fatty acid/ Amine
Cer 17:4 /ceramide 17:4	-1.32	0.033	Ceramide
P-PE 40:8	-1.12	0.041	Phospholipid
P-PE 41:6	0.85	0.049	Phospholipid
PS 45:9 / phosphatidylserine 45:9	1.17	0.002	Phospholipid
PS 40:0 /phosphatidylserine 40:0	1.34	0.004	Phospholipid
1-octadecanoyl-2-(7Z,10Z,13Z,16Z)-docosatetraenoyl-sn-glycero-3-phosphoethanolamine	1.61	0.035	Phospholipid
1-hexadecanoyl-2-(4Z,7Z,10Z,13Z,16Z,19Z-docosahexaenoyl)-sn-glycero-3-phosphocholine	2.78	0.013	Phospholipid
1-[(9Z)-octadecenoyl]-2-[(4Z,7Z,10Z,13Z,16Z,19Z)-docosa-hexaenoyl]-sn-glycero-3-phosphocholine	3.10	0.007	Phospholipid
Platelet-activating factor	6.03	0.015	Phospholipid
1-Linoleoyl-sn-glycero-3-phosphocholine	6.06	0.007	Phospholipid
1-arachidonoyl-sn-glycero-3-phosphocholine	6.10	0.011	Phospholipid
TAG 47:6	-0.68	0.014	Triglyceride
9,12-Hexadecadienoylcarnitine	-2.29	0.002	Acylcarnitine
2-Hexenoylcarnitine	1.43	0.007	Acylcarnitine
stearoylcarnitine	1.62	0.046	Acylcarnitine
3-hydroxydodecanoylcarnitine	2.52	0.014	Acylcarnitine
**DUC trained vs. DUC sed**			
Octatrienal	-1.68	0.000	Fatty aldehyde
1-arachidonoyl-sn-glycero-3-phosphocholine	-4.07	0.039	Phospholipid
1-[(1Z,9Z)-octadecadienyl]-sn-glycero-3-phosphocholine	1.28	0.004	Phospholipid
1-(1Z-hexadecenyl)-sn-glycero-3-phosphocholine	2.46	0.021	Phospholipid
1-[(9Z)-hexadecenoyl]-sn-glycero-3-phosphocholine	4.62	0.006	Phospholipid
Docosanoyl-lysophosphatidylethanolamine	4.71	0.048	Phospholipid
